# The Migration Rules of Malathion during Indoor Simulated Lake Freezing

**DOI:** 10.3390/toxics11030222

**Published:** 2023-02-26

**Authors:** Yan Zhang, Xiaozhuang Wang, Wanli Zhao, Yucan Liu, Tongshuai Liu, Peiyuan Yang

**Affiliations:** 1 College of Civil Engineering, Yantai University, Yantai 264000, China; 2 Logistics Division, Zibo Vocational Institute, Zibo 255000, China

**Keywords:** malathion, ice, icebound, environmental fate

## Abstract

The effect of malathion in ice is a poorly researched area, and ice is an important habitat for organisms at the base of the food web. This study presents laboratory-controlled experiments designed to investigate the migration rule of malathion during lake freezing. Concentrations of malathion were determined in samples of melted ice and in under-ice water. The effects of the initial sample concentration, freezing ratio, and freezing temperature on the distribution of malathion in the ice–water system were investigated. The concentration effect and migration capacity of malathion during freezing was characterized by the concentration rate and distribution coefficient. The results showed that the formation of ice led to the concentration of malathion appearing as follows: concentration in under-ice water > concentration in raw water > concentration in ice. This implied that malathion tended to migrate from the ice to the under-ice water during the freezing process. The increase in the initial malathion concentration, freezing ratio, and freezing temperature caused a more pronounced repulsion of the malathion by the ice and increased the migration to the under-ice water. When the solution of malathion with an initial concentration of 50 μg/L was frozen at –9 °C and the freezing ratio reached 60%, the concentration of malathion in the under-ice water was concentrated to 2.34 times the initial concentration. The migration of malathion to under-ice water during freezing may pose a potential threat to under-ice ecology; therefore, the environmental quality and impact of under-ice water in icebound lakes needs to be given more attention.

## 1. Introduction

Water being icebound is an important hydrological feature at high altitudes and latitudes [1], with more than 50 million lakes worldwide being periodically frozen [2]. Ice cover during the icebound season not only affects light penetration [3] but also reduces the rate of material and energy exchange between the atmosphere and water [4]. The biodegradability and hydrolysis rate are reduced by the lower under-ice water temperature [5,6]. The reoxygenation process [7,8,9], dilution process [10], and photolysis reaction [11] of water are hindered; thus, the self-purification capacity of under-ice water is greatly diminished [12,13]. The polluted characteristics of the lakes during the icebound season have peculiar features. 

In recent years, more and more researchers have focused on the environmental behavior of pollutants during lake freezing. It was found that during water freezing, the concentration of contaminants in under-ice water and sediments increases due to the migration of contaminants into under-ice water and sediments [14,15]. Rapidly increasing concentrations over a short period of time can have a large impact on the ecosystems in under-ice water and disrupt the original ecological balance [16]. When the temperature rises, the pollutants that are trapped in the ice are released into the water [10]. In addition, during water freezing, most of the inorganic salts migrate from the ice to the under-ice water, which is where the concentration of inorganic salts increases. Due to the difference in equilibrium concentration, inorganic salts would then further migrate from the under-ice water into the sediment [17,18,19]. Sun et al. [20] found that inorganic ions in Wuliangsuhai Lake would migrate to the under-ice water in the winter, and the migration of inorganic ions was related to the energy difference between the ice and water as observed using first-principles calculations. During water freezing, nutrient salts also migrate to the under-ice water. Hampton et al. [9] collated and analyzed data from 101 lakes around the world and found that the total dissolved nitrogen and total nitrogen content of water during icebound periods was higher than during non-icebound periods. The increase in nitrogen and phosphorus in the under-ice water during the freezing caused the eutrophication of the water, which led to an increase in chlorophyll and algae [21]. Dissolved organic matter has the same rule in the process of water freezing. Xue and Yang [22] found that, when compared with the organic compounds constituting DOC, UV-absorbing compounds, trihalomethane precursors, and fluorescent materials of fulvic acid samples were more likely to be repelled by the ice phase and were more likely to remain in the unfrozen liquid phase during water freezing.

The environmental behaviors of persistent organic pollutants (POPs) such as the organochlorine drug β-hexachlorocyclohexane [23], polycyclic aromatic hydrocarbons (PHAs) [24], the insecticide chlorpyrifos, and the herbicide artemisinin [25] during the icebound season were studied. The authors found that the concentration of POPs in under-ice water may increase due to the freezing concentration effect. When the ice melted in the spring, the POPs that were frozen in the ice were released back into the water [26,27]. Although melting ice can lead to changes in the concentration of POPs, it is not the only way; other examples are storm water runoff, sewage discharges, etc. [28]. However, the environmental behavior of the organophosphorus-like compound malathion during freezing has not been reported. 

**Figure 1:**
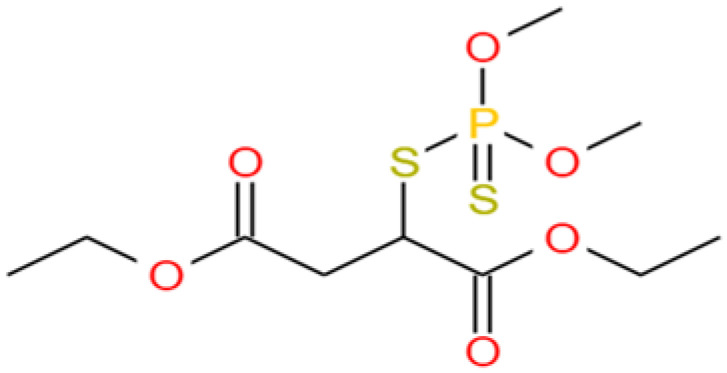
Structural formula of malathion.

Malathion (Figure 1) is an organophosphorus pesticide. After the European Union banned the use of organochlorine pesticides, the amount of malathion usage has gradually increased [29]. The half-life of malathion in water was not fixed and was clearly affected by pH. Malathion degradation in acidic conditions was relatively slow, while malathion degradation in alkaline conditions was sufficiently rapid. Temperature affects the degradation products of malathion [30]. Studies have clearly shown that the half-life of malathion in water at pH = 6.0 was about one to two weeks. The half-life of malathion in alkaline (pH = 8.2) natural rivers was 22 h [31]. Different ionic strengths in the solvation and buffer solution concentrations could affect the degradation of the malathion. Photolysis was observed to be one of the degradation pathways of malathion, but it was not the dominant pathway. Malathion enters the aqueous environment through precipitation, runoff, and infiltration processes, resulting in a high detection rate of organophosphorus pesticides in natural water [32,33,34]. The concentration of malathion in the water environment is not fixed and varies from region to region and with different requirements [35]. Fadaei et al. [36] investigated the distribution and composition of pesticides in the Babolrood River in Mazandaran Province, Iran; the results showed that the average level of malathion in the surface water was 55.7−75.9 μg /L, and the highest level was 506.6 μg /L. According to the specific project standards of surface water source for centralized drinking water in China’s "Surface Water Environmental Quality Standards (GB3838-2002)" and the unconventional water quality standards in "Sanitary Standards for Drinking Water (GB5749-2006)", the concentration of malathion should not exceed 0.05mg/L or 0.25mg/L, respectively. Toxicological studies have shown that trace levels of malathion residues in drinking water are cytotoxic, teratogenic, and mutagenic [37,38]. Trace amounts of malathion can cause endocrine disruption and damage the nervous and immune systems of mammals [39,40]. Excessive use of malathion could lead to resistance in some organisms and disrupt the ecological balance [41,42]. 

It has been shown that the degradation rate of malathion is significantly reduced under low-temperature and dark conditions [43,44]; therefore, if malathion also migrates to under-ice water during freezing, this would pose a great challenge to the ecology of under-ice water. This study focused on the migration rule of malathion during the water freezing process by simulating the freezing process of natural water. Moreover, the effects of freezing temperature, initial malathion concentration, and freezing ratio on the migration of malathion during the freezing process were clarified. The differences in the distribution of malathion in ice meltwater and water under ice were analyzed to understand the rejection behavior of ice crystals to malathion during freezing; this was performed in order to provide theoretical basis and data support for the management of the water environment of lakes during the icebound period.

## 2. Materials and Methods

### 2.1. Experimental Setup

**Figure 2:**
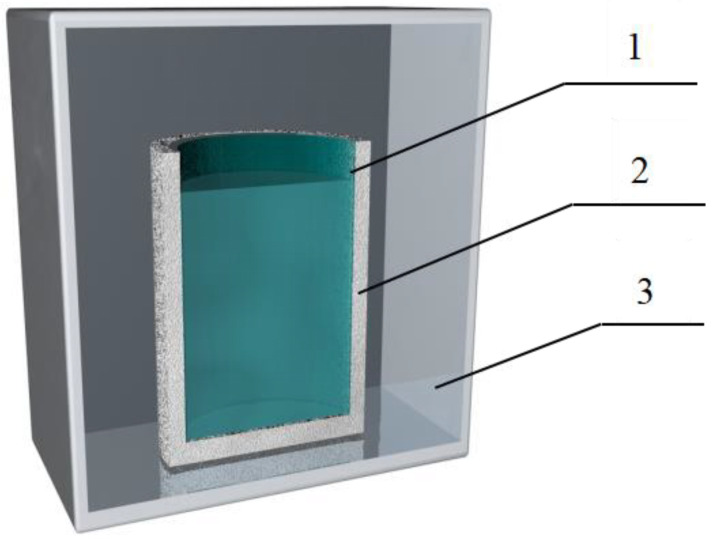
Schematic diagram of the ice simulation experiment device. 1—High borosilicate glass reactor; 2—EPS insulation layer; 3—Low temperature test chamber.

In order to simulate the natural icing process of lake water, an open and unidirectional cooling conduction freezing simulation device was designed (Figure 2). The upper part of the device had an opening for unidirectional cold energy transfer and a built-in high borosilicate glass tank reactor (wall thickness 2 mm, diameter 8 cm, height 21.5 cm). The perimeter and bottom were wrapped with EPS insulation material to block the heat transfer between the container and the outside world. The freezing device was placed in a low-temperature test chamber (BC/BD-519HEX, Haier, Qingdao, China) to perform the freezing experiments. The volume of the low-temperature test chamber was 519 L, and the minimum refrigeration temperature could reach −40 °C.

### 2.2. Instruments and Reagents

The main instruments were an ultra-performance liquid chromatography-electrospray ionization-triple quadrupole mass spectrometer (UPLC-ESI-MS/MS) (Waters, Milford, MA, USA) and ACQUITYTM UPLC BEN C8 column (2.1 mm × 50 mm × 1.7 μm). The instruments were used to detect malathion in samples. The experiment took place at the Micropollution Research Laboratory of Yantai University.

Main reagents: malathion from AccuStandard, purity ≥ 97.9%; methanol, from J&K, HPLC grade, purity ≥ 99.9%; and acetonitrile, from J&K, HPLC grade, purity ≥ 99.9%.

### 2.3. Experimental Design and Procedure

To investigate the migration rule of malathion under different freezing conditions, the three factors initial sample concentration (30 μg/L, 40 μg/L, 50 μg/L, 60 μg/L, and 70 μg/L), freezing ratio (15%, 30%, 45%, and 60%), and freeze temperature (−3 °C, −6 °C, −9 °C, −12 °C, and −15 °C) were set as experimental variables.

A malathion solution (pH = 7.0) of 1 L was prepared in the freezing simulator with ultrapure water according to the desired concentration. The malathion samples were placed in the low-temperature test chamber and under the desired freezing conditions. When the set experimental conditions were reached, the ice and the under-ice water were separated. The ice melted under room temperature conditions. Malathion concentrations were separately measured in under-ice water and ice melt water.

The pH (pH = 7.0) of our solution did not change during the whole experiment. According to the conservation of mass, there was no degradation of malathion during the whole experiment. Our verification of the conservation of mass is shown in Appendix A.

### 2.4. Operating Parameters of Ultra Performance Liquid Chromatography

Mass spectrometry operating conditions: electrospray ionization source (ESI^+^); multiple reaction monitoring (MRM) mode; the collision gas was argon; the desolvention was nitrogen; the ion source temperature was 120 °C; the desolvention temperature was 350 °C; the desolvention flow rate was 300 L/h; and the cone gas flow rate was 50 L/h. The mass spectrometry parameters are shown in Table 1.

Chromatographic operating conditions: mobile phases were methanol and ultrapure water; the column temperature was 35 °C; the sample chamber temperature was 20 °C; and the total flow rate of the multiplex pump was 0.2 mL/min. The flow gradient is shown in Table 2.

### 2.5. Observation of Malathion Distribution in Ice

The surface light pole detection system consists of a light source, 300 W xenon lamp and image acquisition device, and DSLR camera (EOS 5D Mark II, Canon, Japan). To prevent interference from stray light, the equipment was shielded. The malathion ice sample was frozen in a quartz colorimetric container. The side wall and bottom of the container was wrapped with insulating cotton and frozen in the low temperature test chamber. The collected image was converted into a pseudo color standard image with a 16-bit floating point (gray) format through Photoshop (CS6).

### 2.6. Data Analysis Methods

The concentration rate *P* was defined as the ratio of the concentration of malathion in the under-ice water and in the raw sample to characterize the effect of the freezing process on the concentration of malathion.

(1) $$P\;=\;\frac{C_{iw}}{C_{w}}\;\times \;100\%$$

where *C_w_* is the average concentration of malathion in the raw sample and *C_iw_* is the concentration of malathion in the under-ice water.

The distribution coefficient *K* [45] was defined as the ratio of the concentration of malathion in ice to the concentration of malathion in under-ice water to characterize the transport capacity of malathion during freezing.

(2) $$K\;=\;\frac{C_{i}}{C_{iw}}$$

where *C_i_* is the average concentration of malathion in the ice.

## 3. Results

### 3.1. Effect of Initial Sample Concentration on the Migration Rule of Malathion

**Figure 3:**
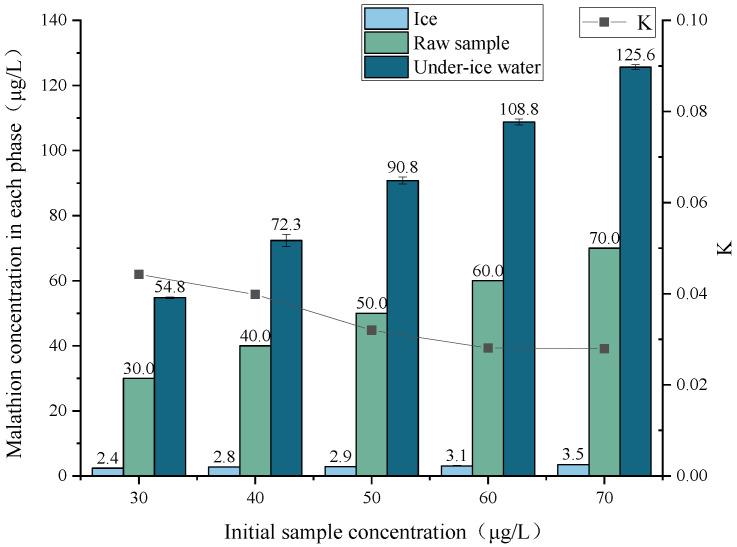
Migration rule for the different initial sample concentrations of malathion.

Malathion solutions with initial sample concentrations of 30 μg/L, 40 μg/L, 50 μg/L, 60 μg/L, and 70 μg/L were prepared and frozen at −9 °C until the freezing ratio was 45% for the separation of ice and water. The concentrations of malathion in the under-ice water and in the ice melt water were separately detected, and the effect of the initial concentration on the migration rule of malathion during the freezing process was analyzed by plotting Figure 3.

As shown in Figure 3, the concentration relationships of malathion in the initial sample concentrations, under-ice water, and ice melt water for each initial sample concentration condition were in the order of: under-ice water > initial solution > ice melt water. Malathion concentrations in both ice and under-ice water exhibited an increase in response to initial sample concentration. The concentration rate *p* values of malathion at the initial sample concentrations of 30 μg/L, 40 μg/L, 50 μg/L, 60 μg/L, and 70 μg/L were 183.0%, 181.0%, 182.0%, 181,0%, and 180.0%, respectively, and the distribution coefficient *K* values were 0.044, 0.040, 0.032, 0.028, and 0.028, respectively. The results showed that as the concentration increases, the rejection of malathion by the ice increases, and the migration of malathion into the under-ice water was enhanced.

### 3.2. Effect of Freezing Ratio on the Migration Rule of Malathion 

**Figure 4:**
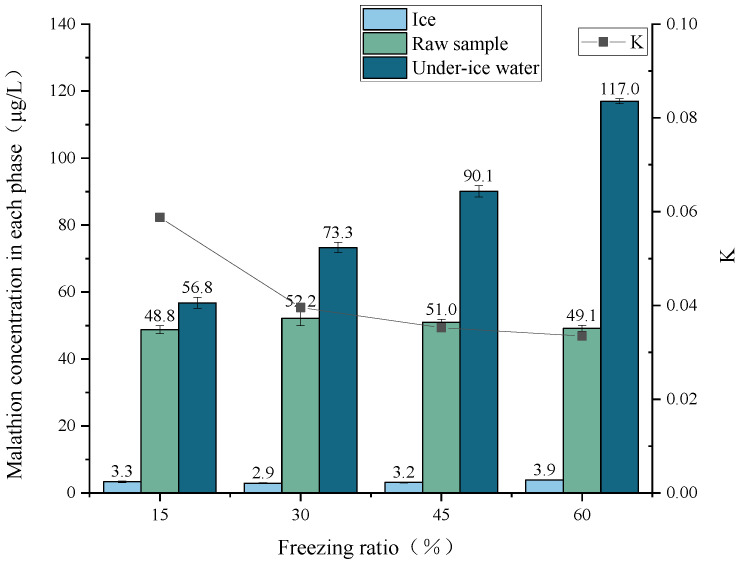
Migration rule of the malathion at different freezing ratios.

Malathion solutions with an initial sample concentration of 50 μg/L were prepared, and freezing experiments were conducted at −9 °C. Ice and water were separated when the freezing ratio reached 15%, 30%, 45%, and 60%. Concentrations of malathion were detected in under-ice water and ice melt water. The effect of the freezing ratio on the migration rule of the malathion during freezing was analyzed by plotting Figure 4.

The concentration of malathion in ice tended to decrease and then increase with the percentage of freezing. The concentration of malathion in the under-ice water was significantly higher than its concentration in the raw sample. The concentration of malathion in the under-ice water showed an increasing trend with increasing freezing ratio. The concentration rate *p* values of malathion (freezing ratio 15%, 30%, 45%, and 60%) were 114.0%, 147.0%, 180.0%, and 234.0%, respectively, and the distribution coefficient *K* values were 0.059, 0.040, 0.035, and 0.033, respectively. The results showed that the rejection of ice from the malathion gradually increases with increasing freezing ratio, and the migration of the malathion to the water below the ice was enhanced.

### 3.3. Effect of Freezing Temperature on the Migration Rule of Malathion 

**Figure 5:**
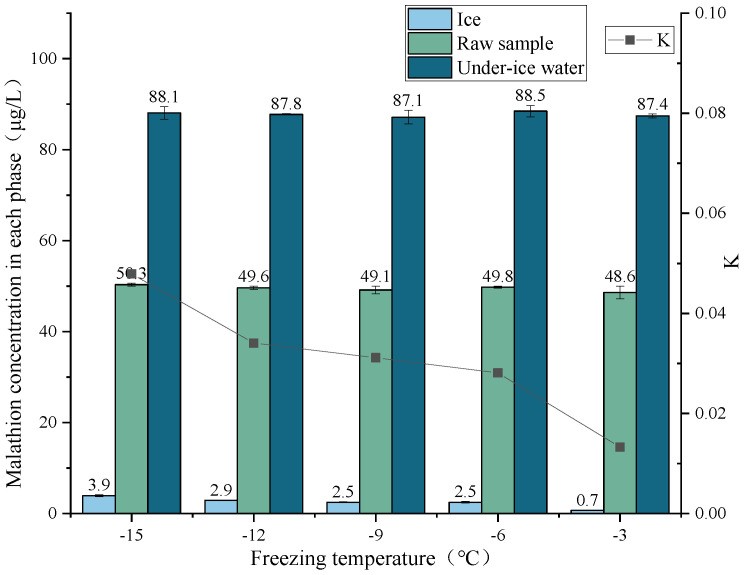
Migration rule of malathion at different freezing temperatures.

Malathion solutions with an initial sample concentration of 50 μg/L were prepared, and freezing experiments were conducted at −15 °C, −12 °C, −9 °C, −6 °C, and −3 °C, respectively. The ice and water were separated when the freezing ratio reached 45%. Concentrations of malathion were detected in the under-ice water and ice melt water. The effect of the freezing temperature on the migration rule of the malathion during freezing was analyzed by plotting Figure 5.

As shown in Figure 5, the malathion ice concentration decreased compared with the raw sample concentration and was more pronounced at higher freezing temperatures, i.e., more malathion was trapped in ice as the freezing temperature decreased. The concentration of malathion in the under-ice water was significantly higher than in its raw sample, but the change in the concentration of malathion in under-ice water with the increase of freezing temperature was less pronounced. The concentration rate *p* values of malathion (freezing temperatures −15 °C, −12 °C, −9 °C, −6 °C, and −3 °C) were 176.0%, 176.0%, 174.0%, 177.0%, and 175.0%, respectively, and the distribution coefficient *K* were 0.048, 0.034, 0.031, 0.028, and 0.013, respectively. The results showed that the rejection of the malathion by the ice became more pronounced with increasing freezing temperature, while the migration of the malathion to the under-ice water became stronger. 

## 4. Discussion

The migration rule of the malathion during the simulated lake freezing indicated that the malathion migrated from the ice to the under-ice water during the freezing at different initial sample concentrations, freezing ratios, and freezing temperatures. Research on atrazine in the freezing process showed that freezing will lead to the increase in its concentration in the under-ice water [46]. The high concentration of malathion in the under-ice water will pose a great threat to the under-ice water ecology. As for the use of the lake as a source of drinking water, it may also have a direct impact on human drinking water safety.

The degradation pathways of malathion in water are hydrolysis, photolysis, and biodegradation. The rate of hydrolysis strongly depends on the temperature. The half-life of malathion increases with the decrease in water temperature. The half-life of malathion in water at 5 °C is 101.93 d [43]. The degradation of malathion is also affected by light. The presence of an ice cover makes the availability of light for under-ice water slightly lower than in the non-icebound period, and as such, photolysis rates are also affected [26]. The study showed that the concentration of malathion did not change after 140 days of darkness [44]. The presence of the ice cover reduced the temperature of the water and availability of light; this caused the safety of malathion to water environment in icebound to become a more serious issue.

**Figure 6:**
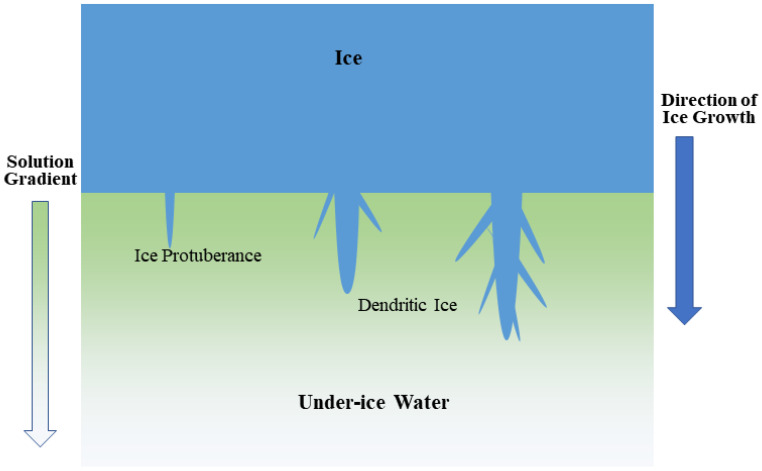
Schematic diagram of the growth of dendritic ice.

The freezing process is the binding of malathion to ice molecules; the binding process releases energy, but the energy released by this process is smaller than that released when malathion binds to water clusters [20]. It is known from quantum chemical theory that the smaller the released energy, the more unstable the chemosynthetic structure becomes. As a result, the malathion tends to bind more strongly to water clusters during freezing. In other words, ice molecules have a rejection effect on the malathion, which concentrates the malathion in under-ice water. However, the natural ice growth process simulated in this study is unidirectional from top to bottom. As the ice growth front proceeds, the thickness of the ice gradually increases, and more and more malathion migrates from the ice to the under-ice water. At the same time, the presence of the ice cover makes the water less disturbed and more stationary. The migration of malathion from the ice to the under-ice water causes a concentration gradient between the ice growth front and the under-ice water, resulting in a "constitutional undercooling" [47]. Constitutional undercooling creates suitable conditions for dendritic ice growth (Figure 6) [48]. The ice growth process was not flat, but rather had a bulge (ice protuberance), where from the bulge the ice grew downward. The bulge formed dendritic ice. As more and more dendritic ice grew, it connected with itself to form ice. However, if the connected dendritic ice was spaced relatively far apart, some malathion was "trapped" in the ice by the growth of dendritic ice during the freezing process [49]. This process makes the concentration of malathion in ice much smaller than it is in the under-ice water.

The process of malathion "captured" by ice is influenced by both the ice growth rate and the malathion diffusion rate. When the ice growth rate is greater than the diffusion rate of malathion, malathion is not able to diffuse and is "captured" by the ice [50]. The freezing temperature is a major factor in the growth rate of ice. As the freezing temperature decreases, the growth rate of ice gradually increases and the amount of malathion "captured" in the ice increases. The process of the ice "capture" of malathion is also related to the contact opportunities between malathion molecules and ice molecules. The increase in the initial concentration of malathion improves the chance of contact with ice molecules and makes it easier for it to be "captured" in the ice. 

**Figure 7:**
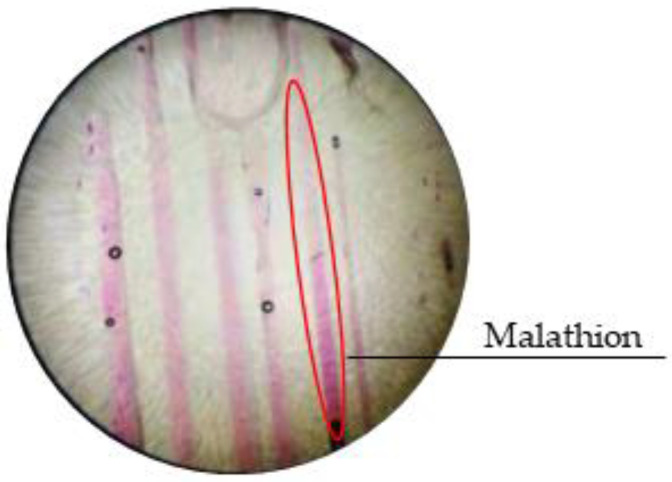
Pseudo-color image of malathion in pure ice.

Through the observation of the distribution of malathion in ice, it can be found that although most of the malathion is expelled into the under-ice water during the process of freezing, a small part of the malathion is still "captured" in the ice (Figure 7). This discovery confirmed that malathion would be "captured" by ice during its growth. If the malathion is also preferentially released, it will have a greater impact on the under-ice water ecology, which is also the focus of future research.

The gradual thickening of the ice cover follows the continued growth of the ice. The thicker ice cover weakens heat transfer, which in turn leads to slower ice growth, meaning that the rate of ice growth decreases as the freezing ratio increases. Therefore, ice crystals can "capture" more malathion when the volume ratio of ice to water is low. However, as the freezing ratio of ice increases, the concentration of malathion in the under-ice water also gradually increases. This increases the contact opportunities between malathion and the ice molecules, which means that the ice can "capture" more malathion. In this study, the concentration of malathion in ice decreased and then increased as the volume ratio of ice to water increased (Figure 4). This indicates that the ice growth rate played a dominant role in the "capture" process in the early stage, while the contact opportunities between malathion and ice molecules played a dominant role in the later stage.

## 5. Conclusions

In this paper, we simulated the freezing process of natural water by laboratory experiments. The effects of the freezing temperature, initial concentration, and freezing ratio on the migration of malathion during the water freezing process were investigated. The distribution of malathion during the icing process was analyzed. The presence of malathion in the ice was discovered by observing the structure of ice. Experiments showed that the ice growth process was not flat, but rather had a bulge; from the bulge, the ice grew downward. The bulge formed dendritic ice. As more and more dendritic ice grew, the dendrites connected with each other to form ice. However, if the connected dendritic ice was spaced relatively far apart, some malathion was "trapped" in the ice by the growth of dendritic ice during freezing. Nevertheless, its concentration in the ice was significantly lower than that of in the water. The distribution coefficient of malathion increased with the increase in initial sample concentration, freezing ratio, and freezing temperature; that is, the migration ability of malathion to under-ice water increased. When the solution of malathion with an initial concentration of 50 μg/L was frozen at –9 °C and the freezing ratio reached 60%, the concentration of malathion in the under-ice water was concentrated to 2.34 times the initial concentration, which is worthy of our attention. For shallow lakes at high latitudes and high altitudes, the proportion of ice in these lakes are high. The concentration of malathion in under-ice water will substantially increase with the proportion of ice. High levels of malathion pose a greater risk to human health and will adversely affect the already fragile under-ice water ecology during icebound periods. This poses new challenges for the management of water environments during the icebound periods of lakes. 

## Figures and Tables

**Table 1:** Parameters of the mass spectrometry MRM detection mode.

Chemical Compound	Mass-To-Charge Ratio (*m*/*z*)		Collision Energy (eV)
	Parent Ion	Daughter Ion	
**Malathion**	330.96	98.98	25
		126.99	13
		285.01	7

**Table 2:** Parameters of the mobile phase in the liquid chromatography analysis.

Time (min)	Flow Rate (mL/min)	Mobile Phase A (%)	Mobile Phase B (%)
Initial	0.200	60.0	40.0
0.20	0.200	60.0	40.0
3.50	0.200	100.0	0.0
3.60	0.200	60.0	40.0
6.50	0.200	60.0	40.0

## Data Availability

Data is available from the corresponding author upon request.

## Appendix A. Mass Conservation Verification

**Table A1:** Mass conservation verification of the freezing ratio.

Freezing Ratio (%)	Raw Sample		Ice		Under-Ice Water		CHECK IN/OUT (%)
	Concentration (μg/L)	Volume (L)	Concentration (μg/L)	Volume (L)	Concentration (μg/L)	Volume (L)	
15	48.8	1	3.3	0.15	56.8	0.85	99.95
30	52.2	1	2.9	0.3	73.3	0.7	99.96
45	51	1	3.2	0.45	90.1	0.55	99.99
60	49.1	1	3.9	0.6	117	0.4	100.08

**Table A2:** Mass conservation verification of the initial concentration.

Initial Concentration (μg/L)	Raw Sample		Ice		Under-Ice Water		CHECK IN/OUT (%)
	Concentration (μg/L)	Volume (L)	Concentration (μg/L)	Volume (L)	Concentration (μg/L)	Volume (L)	
30	30	1	2.4	0.45	54.8	0.55	104.07
40	40	1	2.8	0.45	72.3	0.55	102.56
50	50	1	2.9	0.45	90.8	0.55	102.49
60	60	1	3.1	0.45	108.8	0.55	102.06
70	70	1	3.5	0.45	125.6	0.55	100.94

**Table A3:** Mass conservation verification of the freezing temperature.

Freezing Temperature (°C)	Raw Sample		Ice		Under-Ice Water		CHECK IN/OUT (%)
	Concentration (μg/L)	Volume (L)	Concentration (μg/L)	Volume (L)	Concentration (μg/L)	Volume (L)	
−15	50.3	1	3.9	0.45	88.1	0.55	99.82
−12	49.6	1	2.9	0.45	87.8	0.55	99.99
−9	49.1	1	2.5	0.45	87.1	0.55	99.86
−6	49.8	1	2.5	0.45	88.5	0.55	100.00
−3	48.6	1	0.7	0.45	87.4	0.55	99.56
